# Nasal polyps with osseous metaplasia: A misunderstood situation

**DOI:** 10.1002/ccr3.2945

**Published:** 2020-05-22

**Authors:** Quentin Mat, Rupal Mehta, Sophie Tainmont, Jean‐Pierre Duterme

**Affiliations:** ^1^ Department of Otorhinolaryngology C.H.U. Charleroi Charleroi Belgium

**Keywords:** heterotopic, metaplasia, nasal polyps, ossification

## Abstract

Osseous metaplasia in nasal polyps is rare but benign. To exclude dangerous lesions, sending the entirety of histological samples is mandatory in cases presenting with clustered densities on CT scan. Microdebrider should not be used for this surgery.

## Graphical abstract

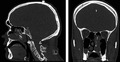

## 1 INTRODUCTION

Osseous metaplasia is the development of normal bone tissue in soft tissue.^1^ Although ectopic bone formation has been well described in gastrointestinal and uterine polyps, this situation remains very unusual in nasal polyps.^1^, ^2^, ^3^, ^4^, ^5^, ^6^ Indeed, only ten cases have been reported in the English medical literature.^1^, ^2^, ^3^, ^4^, ^5^, ^6^ Osseous metaplasia pathophysiology is unknown. Initially, it was thought that this disease originated from a previous surgery. Thus, the new bone formation might arise from bony remnants left behind during prior intervention.^7^ Currently, it has been shown that an overexpression of bone morphogenetic proteins (BMPs) and transforming growth factor β‐1 (TGF β‐1) occurs in mucosal nasal polyps and induces an osteogenic signal in stromal cells.^2^, ^3^, ^5^, ^6^ The aim of this case report is to enable physicians to be aware of this exceptional pathology and to be able to integrate it into their differential diagnosis with more aggressive pathologies.

## 2 CASE REPORT

**Figure 1:**
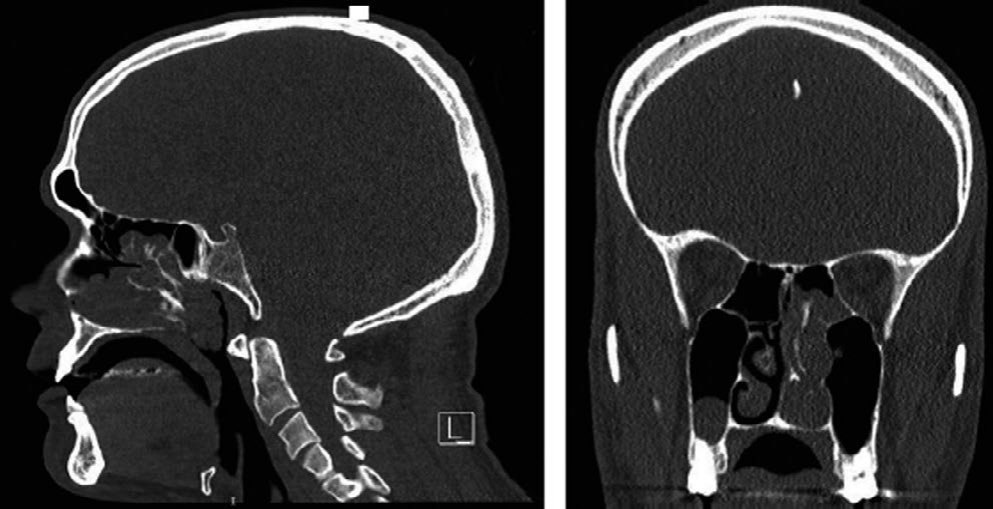
Noncontrast coronal and sagittal CT scans showing central clustered densities inside the posterior ethmoidal cells and the left nasal cavity. These structures are embedded in a polypoid tissue that extends from de left nasal cavity to nasopharynx and left posterior ethmoidal cells

**Figure 2:**
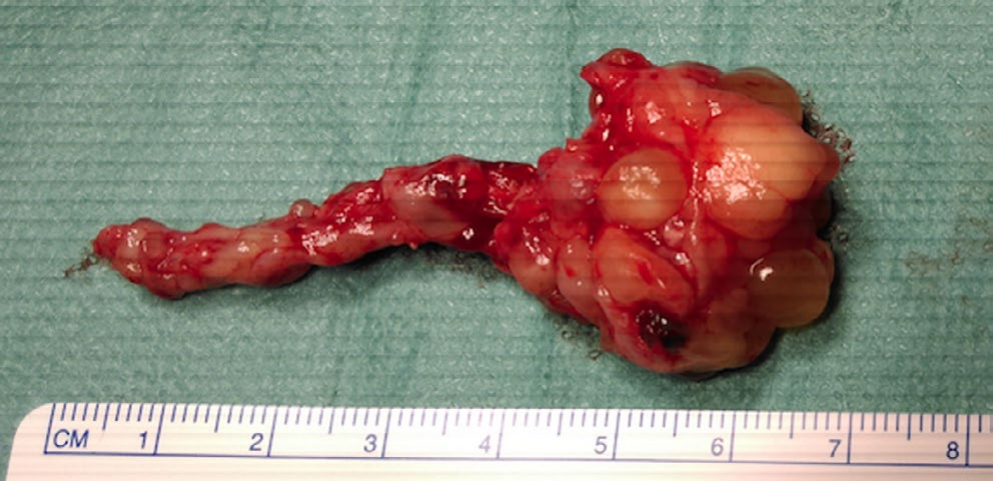
Most important resected polyp with osseous metaplasia

**Figure 3:**
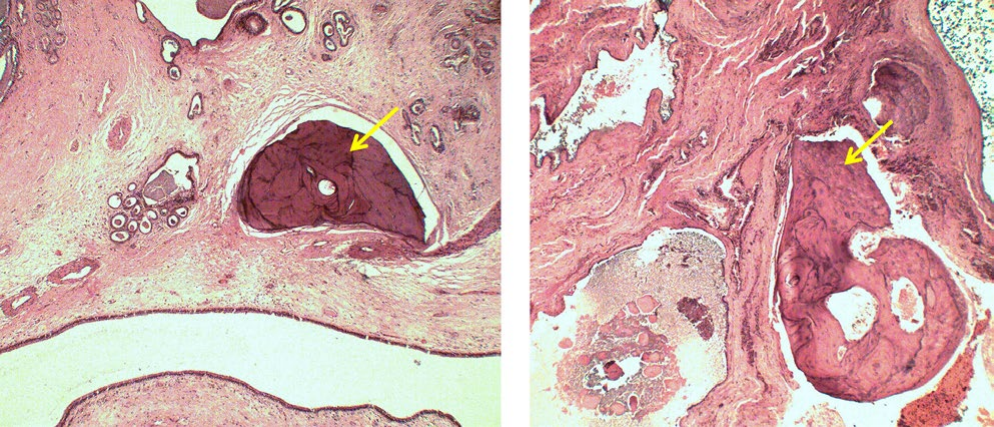
Histopathology revealing an edematous stroma with mature lamellar bone tissue (arrow) covered by respiratory epithelium. Hematoxylin‐eosin, original magnification ×50 on the left and ×100 on the right

A 39‐year‐old Caucasian man was admitted to our hospital with complaints of left‐sided nasal obstruction and anterior rhinorrhea for 2 years. He did not have any other symptoms. The patient had a history of grass and birch pollen allergy which was confirmed by skin prick tests several years ago and was not treated. He did not undergo prior nasal surgery and did not present any peculiar antecedents. Fiberoptic examination revealed a complete obstruction of the left nasal cavity and the nasopharynx by several polyps which occupied the middle and superior meatus as well. Computed tomography scans showed a complete filling of the left nasal cavity that extended into nasopharynx and the left posterior ethmoidal cells. Some central clustered densities were also described in the ethmoid and nasal fossa. There was no bone erosion (Figure 1). Systemic and topical corticosteroids did not have any effect. Therefore, the patient underwent standard endoscopic sinus surgery during which bone fragments were identified inside some polyps (Figure 2). Histopathological examination confirmed the presence of inflammatory polyps with osseous metaplasia (Figure 3). Two years after surgery, the patient presents no recurrence.

## 3 DISCUSSION

While squamous metaplasia is frequent in nasal polyps,^2^, ^3^, ^4^, ^5^ metaplastic ossification is a very uncommon but benign situation.^1^, ^2^, ^3^, ^4^, ^5^, ^6^ Interestingly, the ten cases previously reported are characterized by unilateral location and no resurgence has been mentioned so far.^1^, ^2^, ^3^, ^4^, ^5^, ^6^

None of these patients benefited from an earlier sinonasal surgery.^1^, ^2^, ^3^, ^4^, ^5^, ^6^ This observation strengthens the hypothesis that metaplastic ossification might result from an overexpression of BMPs and TGF β‐1 and thus would induce an osteogenic signal on pluripotential cells that are present in the stroma of mucosal polyps. They could then differentiate into osteoblastic progenitors.^1^, ^2^, ^3^, ^5^, ^6^ It is also possible that there is a dedifferentiation of the stromal cells into pluripotential cells before transforming into osteoblastic progenitors.^2^, ^3^, ^5^, ^6^

The differential diagnosis of sinonasal pathologies with highly radiodense materials includes rhinolith, mycetoma, inverted papilloma with calcifications, chondrosarcoma, osteosarcoma, fibro‐osseous lesions, and seldomly sinonasal adenocarcinoma with metaplastic ossification.^1^, ^3^, ^5^, ^8^ The absence of epistaxis and bone lysis on CT scan are two important aspects that can help clinicians for differentiating harmless etiologies from more aggressive one.^5^ Magnetic resonance imaging is advised when inverted papilloma or cancerous lesions are suspected.^5^ Histological analysis is essential for definitive diagnosis of osseous metaplasia and is characterized by an inflamed and edematous stroma witch contains mature lamellar bone tissue, covered by respiratory epithelium.^5^

## 4 CONCLUSION

Osseous metaplasia in nasal polyps is a unique and benign disease that seems to be characterized by a unilateral location. Contrary to nasal polyposis, it does not tend to relapse after a complete surgical removal. Endoscopic sinus surgery is the only treatment currently recommended. To exclude potentially dangerous lesions and confirm the diagnosis, sending the entirety of histological samples is mandatory in cases presenting with clustered densities on CT scan. For this reason, microdebrider should not be used for this surgery.

## CONFLICT OF INTEREST

The authors have no conflict of interest.

## AUTHOR CONTRIBUTIONS

QM: wrote the case report. All authors read and approved the final manuscript.

## ETHICAL APPROVAL

The scientific publication of this clinical case was approved by the ethics committee of the University Hospital Center of Charleroi.

## CONSENT FOR PUBLICATION

Written informed consent was obtained from the patient for the publication of this report and any accompanying.
